# Pre- and early postpartum psychosocial stress trajectories in mothers and child body mass index at 3 years: a birth cohort study

**DOI:** 10.1186/s12887-023-03991-6

**Published:** 2023-04-15

**Authors:** Stefanie Braig, Deborah Kurz, Martin Wabitsch, Frank Reister, Jon Genuneit, Dietrich Rothenbacher

**Affiliations:** 1 Institute of Epidemiology and Medical Biometry, Ulm University, Helmholtzstraße 22, 89081 Ulm, Germany; 2 Division of Pediatric Endocrinology and Diabetes, Department of Pediatrics and Adolescent Medicine, Ulm University Medical Center, Ulm, Germany; 3 Department of Gynecology and Obstetrics, University Medical Center Ulm, Ulm, Germany; 4 Pediatric Epidemiology, Department of Pediatrics, Medical Faculty, Leipzig University, Leipzig, Germany

**Keywords:** Maternal stress, Maternal depression symptoms, Maternal anxiety symptoms, Child weight, Trajectories

## Abstract

**Background:**

Child overweight remains a prevalent public health concern, but the impact of maternal psychosocial stress and related constructs, the timing, and possible trajectories on child body mass index (BMI) is controversial. We aimed to investigate the association of maternal stress, depression and anxiety symptoms, and maternal hair cortisol concentrations (HCC) at delivery, 6, and 12 months postpartum with child BMI and age- and sex-standardized BMI (BMI-SDS) at age 3 years.

**Methods:**

Data were derived from the Ulm SPATZ Health Study with a baseline examination between 04/2012 and 05/2013 at the University Medical Centre Ulm, Germany, the only maternity clinic in Ulm, with a good representation of the source population. Adjusted regression analyses based on BMI/BMI-SDS (dependent) and trajectories of stress, depression, and anxiety (independent variables) were investigated in 596 mothers and children. Multiple imputation of missing covariates was performed.

**Results:**

Various trajectories in independent variables were identified, trajectories of maternal anxiety symptom differed between child sexes. We did not find an association between trajectories of maternal chronic stress, depression symptoms, or HCC and child BMI/BMI-SDS. However, trajectories of low-increasing maternal anxiety symptoms were linked to higher child BMI compared to a low-stable trajectory group (b = 0.58 kg/m^2^, 95% Confidence Interval: 0.11; 1.04) in girls.

**Conclusions:**

Trajectories of maternal anxiety symptoms were associated with the child’s BMI/BMI-SDS in girls at age 3 years. However, further large scale studies should include variables to determine the causal pathway and enlighten sex-specific differences.

## Background

Among adolescents who are obese, the greatest increase in body mass index (BMI) occurred between 2 and 6 years of age and BMI at age 3 is a very good predictor of BMI in later ages [1]. Therefore early interventions to prevent overweight and obesity in young children are needed. Nevertheless, while multiple contributors to child BMI have been discovered (e.g., details in review [2]), the impact of maternal psychosocial stress as well as of symptoms of anxiety and depression, its timing and duration on the outcome remains unclear. Especially the association of maternal mental health trajectories with child’s BMI is rarely investigated, yet. Even though it is estimated that the prevalence of maternal depression in the peripartum period is as high as 11.9% (95% Confidence Interval (CI) 11.4–12.5%) [3] and that of any anxiety disorders 15.2% (95% CI 9.0–21.4) [4], both worldwide, often recurring later in maternal life.

Several mechanisms by which maternal psychosocial stress as well as anxiety and depression symptoms might affect child BMI have been proposed, see e.g., [5] including low responsiveness to child’s needs in highly stressed mothers, extensive controlling of the child’s feeding behaviours and modelling of unhealthy life style [6, 7] in children. Furthermore, high levels of maternal stress may negatively impact the mother–child relationship, which influences the child’s obesity risk [8]. Additionally, postpartum depression has been associated with reduced breastfeeding duration [9].

In a meta-analysis of 17 studies investigating the association between *maternal psychosocial stress* and child BMI, the overall effect size was positive, with significant heterogeneity among studies [10]. Yet, one more recent review does not support an association between psychosocial maternal stress measured at multiple time points in the first 1000 days and child weight [11]. Moreover, a further review with a focus on *maternal depression* or *depressive symptoms* summarized that chronic but not episodic maternal depression is associated with a greater risk for child overweight [12]. Similarly, no association between maternal depression (not further specified in duration) and child BMI was observed in a more recently conducted Dutch study [13] and in an US population, further adjusted for multiple covariates such as breastfeeding duration [14]. It was hypothesized that pre- and postpartum maternal depression might be differentially associated with child weight [15]. Concerning *maternal anxiety*, evidence of a relationship with child weight is scarce, [16] particularly data describing the association with multiply assessed symptoms of anxiety.

The aim of our study therefore was to analyze the potential associations between trajectories of (1) maternal chronic stress and (2) trajectories of symptoms of anxiety and depression during pregnancy and beyond with child BMI and BMI-SDS (age- and sex-standardized BMI according to a German population). In detail, we aimed to investigate how timing and trajectories of maternal psychosocial stress, depression and anxiety symptoms at delivery, age 6 and 12 months postpartum are associated with child BMI/BMI-SDS at age 3 years taking into account potential covariates. We hypothesized that analyzing trajectories instead of single time points might be advantageous to detect potential associations and that the effects of exposure variables might differ. Moreover, we estimated trajectories of maternal hair cortisol concentrations (HCC) and their associations with child BMI assuming that HCC might be an aggregated surrogate marker of maternal stress exposure.

## Methods

### Study design and study population

**Fig. 1:**
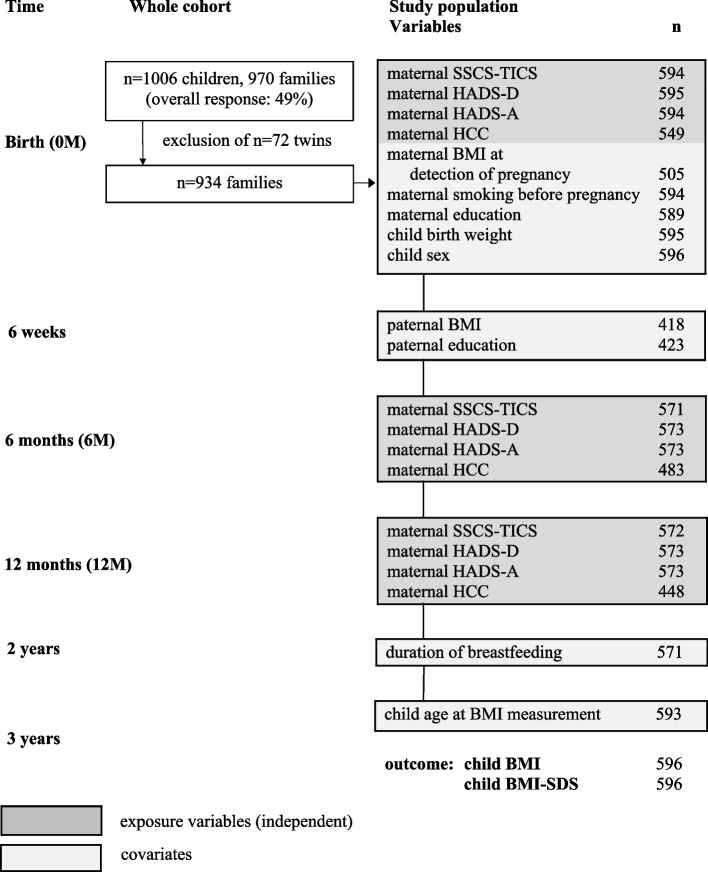
Flowchart. SSCS-TICS Maternal chronic stress, HADS-D Maternal depression symptoms, HADS-A Maternal anxiety symptoms, HCC Hair cortisol concentrations, BMI Body mass index, BMI-SDS age- and sex-standardized BMI

Data were derived from the Ulm SPATZ Health Study, a birth cohort study, conducted in Ulm, Germany. Families in whom the mothers gave birth to a child at Ulm University Medical Centre from 04/2012 to 05/2013 were asked to participate. Participation was 49%, *n* = 970 mothers, 1006 children − among them 934 mothers with singleton children. The Department of Gynaecology and Obstetrics at Ulm Medical Centre is the only maternity clinic in Ulm with a good representation of the source population. Details of the baseline examination were described elsewhere [17]. The study population comprised all mothers with their singleton children for whom data on at least one exposure variable and on the outcome (*n* = 596) were available (for further information on the recruiting process and missing values see Fig. 1). The study was approved by the ethics board of Ulm University (No. 311/11). All participants gave written informed consent. Informed consent was also obtained from the parent and/or legal guardian of minors. The study was performed in accordance with the Declaration of Helsinki.

### Exposure variables

At delivery (0 M), at 6 (6 M) and 12 months (12 M) postpartum, chronic maternal stress was measured using the Screening Scale of the Trier Inventory of Chronic Stress (SSCS-TICS) [18] included in a self-administered standardized questionnaire. SSCS-TICS was developed to assess chronic concerns, lacking social recognition, work overload, excessive demands, and social stress three months prior to assessment. Furthermore, the German version of Hospital Anxiety and Depression Scale (HADS), referring to symptoms of anxiety and depression in the last week prior to completion of the questionnaire was used [19]. Summary scores of eleven or more in each subscale indicate moderate to severe, scores between 8 and 10 mild symptoms, and scores < 8 are considered normal. Hair samples were collected from the posterior vertex region on the maternal head by trained study staff (at 0 M) or by the families themselves (at 6 M and 12 M). HCC were determined in the scalp-near 3 cm hair segment, assuming to reflect cumulative steroid secretion over the period of the preceding 3 months. Further details including the analytical process were described in details elsewhere [17, 20]. HCC values of < 0.09 and > 90 were excluded, [20] so were values > 3 standard deviations from the mean [21].

### Outcome

Child body height and weight measured at the 3-year-screening examination (child age 36.0 months, standard deviation: 1.1) was transcribed by the mothers from the child’s paper-based medical record (“Kinderuntersuchungsheft”), which is started at birth (day 3 to 10) and includes up to 9 screening exams up to age 9 years. Height and weight were measured by the paediatrician, normally including clothing but not shoes. However, the information whether the child was dressed during the measurement was not given in detail. The outcome was the body-mass index (BMI (height/weight^2^)) and the age- and sex-standardized BMI according to a German population [22]. For this purpose, missing age at BMI measurement was set to 3 years (36 months) in *n* = 3 participants corresponding to the mean of the remaining study population. For descriptive findings BMI was classified into underweight or normal, overweight, and obese based on < 90^th^ percentile (P), ≥ P90 – < P97, ≥ P97 of a German population. The classification according to the International Obesity Task Force (IOTF) [23], and the World Health Organization (WHO) [24] was used additionally. All three classifications are age- and sex-specific.

### Covariates

Variables describing the study population such as maternal age (≤ 25, 26–35, ≥ 36 years), number of persons in the household at delivery (2–3, 4, > 4 persons), nationality (derived from nationality and place of birth), maternal education (< 12, ≥ 12 years), smoking during pregnancy (yes, no), and smoking in the year before pregnancy (yes, no) were collected following delivery using a self-administered standardized questionnaire. We used smoking before pregnancy as covariate due to a possible underreporting of smoking during pregnancy. Maternal BMI was calculated based on mother-reported height and physician-recorded measured maternal body weight. The latter was obtained from paper documentation of routine preventive examination that obstetricians issue to their patients when pregnancy is clinically established. However, if first examination took place at > 90 days of gestation, we used self-reported maternal body weight assessed when pregnancy was established (*n* = 96). Maternal BMI was classified into underweight or normal, overweight, and obese corresponding to the related BMI < 25.00, 25.00–30.00, > 30.00 kg/m^2^ for purpose of description whereas for regression models, maternal BMI was used continuously. Child birth weight was drawn from the clinic documentation system. For regression models, child birth weight was used continuously. The following categories were used for description < 2500, 2500– < 3000, 3000– < 3500, 3500– < 4000, ≥ 4000 g. Breastfeeding duration (days) was mother-reported via self-administered standardized questionnaire at a 6-week-, 6 M-, 12 M-, and the 24 M-examination and considered until the mother had reported that she had completely stopped breastfeeding. Further paternal covariates were considered as follows: Paternal BMI was calculated based on father-reported height and weight 6 weeks after child birth. At this time point, information on paternal education (< 12, ≥ 12 years) was further collected in a self-administered questionnaire.

### Statistics

We describe study characteristics and compare them to the baseline population by indicating 95% CI of the binomial distributions. Measures of central tendency are given for maternal stress as well as depression and anxiety symptoms, and HCC at 0 M, 6 M, and 12 M, including Spearman correlation coefficients. Sex-stratified analyses were performed. We further show depression and anxiety symptoms categorized into relevant groups as suggested [25]. We identified latent class mixed model trajectory groups for chronic stress, depression and anxiety symptoms as well as HCC separately. The appropriate number of trajectory groups was determined by increasing it from 1 to 4 and subsequently staying with the number with the lowest Bayesian Information Criterion (BIC), given that the group size was ≥ 10. For HCC for example, BIC was in favor of 3 groups, but one consisted of only one person. The procedure above was also applied for sex-stratified analyses to examine if a sex-stratified analysis would fit better.

Apart from the trajectory group identified based on HCC, the groups are labelled by their starting point (e.g., low) and the course of symptoms (e.g. increasing; resulting in low-increasing). The impact of trajectory groups on child BMI/BMI-SDS was analyzed by linear regression analysis using the probability of group membership as estimated by the linear mixed model procedure as independent variable. Besides the sex-stratified analysis to determine BIC as outlined above, we evaluated a possible multiplicative interaction between maternal stress and related symptoms trajectories and child sex on child BMI and thus performed sex-stratified analyses for maternal anxiety symptom trajectories (p(interaction) = 0.06). For analyses, maternal BMI at first detection of pregnancy, child birth weight, maternal education, maternal smoking before pregnancy, child sex, and child age at BMI measurement were classified as potential confounders based on literature. So was paternal BMI and paternal education. Breastfeeding duration was not statistically significant associated with the exposure variables or the outcome in bivariate (sex-stratified) regression analyses (*p* > 0.05). Analyses were performed by SAS (Statistical Analysis Software 9.4, SAS Institute Inc, North Carolina, USA). The linear mixed model procedure was calculated by using R (R Foundation for Statistical Computing, Vienna, Austria).

### Missing values

Missing values for the exposure variables were considered as missing at random and were estimated using the R lcmm function. Missing values of the confounders were imputed using PROC MI. All variables were considered as either continuous or were coded as dummy variables. For numbers of the respective missing values see Fig. 1. For analyses, 10 completed data sets were generated and combined in a final step. We included all confounders in our imputation model, all exposures, and the outcome variables as potential predictors of missing values. Two separate imputation models were calculated, one for BMI and one for BMI-SDS. We compared the results between multiple imputation data and the complete case approach.

## Results

Following restriction to singleton full-term deliveries (96.3% of mothers of total), information for 596 mothers and their children was available for this study. Only a small minority (7.1%) was of non-German nationality (see Table 1). Educational level was high and 66.6% of mothers reported ≥ 12 school years. Only 3.4% of mothers reported smoking during pregnancy, while 20.7% reported smoking before pregnancy. Children’s sex was almost equally distributed in the study population (50.7% male). Sex-stratified analyses revealed statistically significant higher maternal BMI in girls as opposed to boys and higher child birth weight. At age 3 years, a total of 5.7% of children were classified as being overweight and 1.8% as obese with reference to a German population, 5.5% and 0.5% with reference to the International Obesity Task Force, and 12.4% and 3.5% with reference to the World Health Organization, respectively (these data of the reference populations are age- and sex-specific). Table 1 also shows the population characteristics of the whole study cohort. Notably, especially the non-German participants were under-, mothers with ≥ 12 school years were overrepresented in the analysis population compared to the baseline population.

**Table 1:** Population characteristics in the Ulm SPATZ health study

	**Study population** **(*N* = 596)**		**Whole cohort** **(*n* = 934)**		
	**n/N**	**%**	**n/N**	**%**	**95% Confidence interval**
**Maternal characteristics assessed at delivery**					
Maternal age [years]					
≤ 25	18/596	3.0	60/933	6.4	4.9; 8.0
26–35	418/596	70.1	642/933	68.8	65.8; 71.8
≥ 36	160/596	26.9	231/933	24.8	22.0; 27.5
Number of persons in household					
2–3	348/594	58.6	501/920	54.5	51.2; 57.7
4	194/594	32.7	306/920	33.3	30.2; 36.3
> 4	52/594	8.8	113/920	12.3	10.2; 14.4
Maternal nationality					
Non-German	42/595	7.1	103/926	11.1	9.1; 13.2
German	553/595	92.9	823/926	88.9	86.9; 90.9
Maternal education [years]					
< 12	197/589	33.5	370/915	40.4	37.3; 43.6
≥ 12	392/589	66.6	545/915	59.6	56.4; 62.7
Smoking during pregnancy					
Yes	20/589	3.4	70/914	7.5	5.9; 9.4
No	569/589	96.6	844/914	92.3	90.6; 94.1
Smoking before pregnancy					
Yes	123/594	20.7	253/922	27.4	24.6; 30.4
No	471/594	79.3	669/922	72.6	69.7; 75.4
Maternal BMI [kg/m^2^]					
Underweight or normal (BMI < 25.00)	333/505	65.9	504/784	64.3	60.9; 67.6
Overweight (BMI 25.00–30.00)	116/505	23.0	183/784	23.3	20.4; 26.3
Obese (BMI > 30.00)	56/505	11.1	97/784	12.4	10.1; 14.7
**Child characteristics**					
Child sex					
Boys	302/596	50.7	494/934	52.9	49.7; 56.1
Girls	294/596	49.3	440/934	47.1	43.9; 50.3
Child birth weight [grams]					
< 2500	31/595	5.2	50/933	5.4	3.9; 6.8
2500– < 3000	97/595	16.3	155/933	16.6	14.2; 19.0
3000– < 3500	247/595	41.5	386/933	41.4	38.2; 44.5
3500– < 4000	170/595	28.6	277/933	29.7	26.7; 32.6
≥ 4000	50/595	8.4	65/933	7.0	5.3; 8.6
Child BMI at age 3 years ^a^					
Underweight or normal (≤ P90)	551/596	92.4	-	-	-
Overweight (> P90-P97)	34/596	5.7	-	-	-
Obese (> P97)	11/596	1.8	-	-	-
Child BMI at age 3 years (IOTF) ^b^					
Underweight or normal	560/596	94.0	-	-	-
Overweight	33/596	5.5	-	-	-
Obese	3/596	0.5	-	-	-
Child BMI at age 3 years (WHO) ^c^					
≤ P85	501/596	84.0	-	-	-
> P85-P97	74/596	12.4	-	-	-
> P97	21/596	3.5	-	-	-

*BMI* Body mass index, *Std* Standard deviation

^a^classification was done based on < 90^th^ percentile (P), ≥ P90 – < P97, ≥ P97 of a German population

^b^International Obesity Task Force

^c^World Health Organization

As displayed in Table 2, median chronic stress (SSCS-TICS) at delivery was 50.0 points (P25: 43.0, P75: 56.0), median HADS-D score: 2.0 points (1.0, 3.0), and median HADS-A score was 4.0 points (2.0, 6.0). The respective values for HCC as potential biological surrogate marker of chronic stress was median: 5.4 pg/mg (3.2; 10.3). Correlation between the different time points was higher in SSCS-TICS (Spearman correlation coefficient *r* = 0.59 (0 M and 6 M), *r* = 0.55 (0 M and 12 M), and *r* = 0.70 (6 M and 12 M)) than in HADS-D (*r* = 0.45, *r* = 0.41, *r* = 0.70)) and HADS-A (*r* = 0.50, *r* = 0.47, *r* = 0.71)). The correlations were notably high between the 6 and 12 months scores. However, low correlations were seen between SSCS-TICS, maternal anxiety or depression symptoms and HCC (*r* ≤ 0.09 at any time point).

**Table 2:** Maternal chronic stress, depression and anxiety symptoms, and hair cortisol concentrations in the Ulm SPATZ health study

	**Time**	**N**	**Mean (Std)**	**Median (P25; P75)**	**Spearman correlation coefficient**		
					**SSCS-TICS**		
					**0 M**	**6 M**	**12 M**
**SSCS-TICS [points] *total***	0 M	594	48.8 (9.7)	50.0 (43.0; 56.0)	1.00	-	-
	6 M	571	47.4 (9.7)	47.0 (42.0; 53.0)	0.59	1.00	-
	12 M	572	47.4 (10.2)	47.0 (42.0; 54.0)	0.55	0.70	1.00
***Child sex: male***	0 M	301	49.2 (9.5)	50.0 (43.0; 56.0)	1.00	-	-
	6 M	288	47.7 (9.7)	49.0 (41.5; 54.0)	0.60	1.00	-
	12 M	286	47.7 (10.2)	48.0 (42.0; 54.0)	0.51	0.70	1.00
***Child sex: female***	0 M	293	48.3 (9.9)	50.0 (42.0; 54.0)	1.00	-	-
	6 M	283	47.1 (9.8)	47.0 (42.0; 53.0)	0.57	1.00	-
	12 M	286	47.2 (10.3)	47.0 (42.0; 54.0)	0.59	0.70	1.00
					**HADS-D**		
**HADS-D [points] *total***	0 M	595	2.4 (2.1)	2.0 (1.0; 3.0)	1.00	-	-
	6 M	573	2.8 (2.5)	2.0 (1.0; 4.0)	0.45	1.00	-
	12 M	573	2.9 (2.5)	2.0 (1.0; 4.0)	0.41	0.70	1.00
***Child sex: male***	0 M	301	2.6 (2.2)	2.0 (1.0; 4.0)	1.00	-	-
	6 M	290	2.9 (2.4)	2.0 (1.0; 4.0)	0.44	1.00	-
	12 M	287	2.9 (2.5)	2.0 (1.0; 4.0)	0.39	0.71	1.00
***Child sex: female***	0 M	294	2.1 (1.9)	2.0 (1.0; 3.0)	1.00	-	-
	6 M	283	2.6 (2.5)	2.0 (1.0; 3.0)	0.42	1.00	-
	12 M	286	2.8 (2.7)	2.0 (1.0; 4.0)	0.41	0.68	1.00
					**HADS-A**		
**HADS-A [points]* total***	0 M	594	4.4 (3.1)	4.0 (2.0; 6.0)	1.00	-	-
	6 M	573	4.8 (3.0)	4.0 (3.0; 7.0)	0.50	1.00	-
	12 M	573	4.8 (3.1)	4.0 (2.0; 6.0)	0.47	0.71	1.00
***Child sex: male***	0 M	301	4.6 (3.0)	4.0 (2.0; 6.0)	1.00	-	-
	6 M	290	5.0 (3.0)	5.0 (3.0; 7.0)	0.52	1.00	-
	12 M	287	4.9 (2.9)	5.0 (3.0; 7.0)	0.49	0.72	1.00
***Child sex: female***	0 M	293	4.3 (3.1)	4.0 (2.0; 6.0)	1.00	-	-
	6 M	283	4.5 (3.0)	4.0 (2.0; 6.0)	0.47	1.00	-
	12 M	286	4.7 (3.4)	4.0 (2.0; 6.0)	0.44	0.68	1.00
					**HCC**		
**HCC [pg/mg] *total***	0 M	549	8.3 (7.7)	5.4 (3.2; 10.3)	1.00	-	-
	6 M	483	5.4 (4.6)	3.7 (2.7; 6. 1)	0.11	1.00	-
	12 M	448	4.9 (4.3)	3.5 (2.4; 5.7)	0.27	0.39	1.00
***Child sex: male***	0 M	276	8.2 (7.5)	5.4 (3.2; 10.6)	1.00	-	-
	6 M	244	5.9 (5.0)	4.0 (2.8; 6.9)	0.15	1.00	-
	12 M	226	5.5 (4.9)	3.8 (2.5; 6.6)	0.34	0.42	1.00
***Child sex: female***	0 M	273	8.3 (7.9)	5.5 (3.1; 9.6)	1.00	-	-
	6 M	239	4.8 (4.0)	3.7 (2.5; 5.5)	0.05	1.00	-
	12 M	222	4.3 (3.6)	3.2 (2.2; 4.9)	0.19	0.34	1.00

*0 M* Delivery, *6 M* 6 months, *12 M* 12 months, *Std* Standard deviation, *P* Percentile, *SSCS-TICS* Maternal chronic stress symptoms, *HADS-D* Maternal depression symptoms, *HADS-A* Maternal anxiety symptoms, *HCC* Hair cortisol concentrations

A categorization of maternal depression and anxiety symptoms according to relevant cut-off points reflected a low level of symptoms of mood disorders (see Table 3).

**Table 3:** Maternal depression and anxiety symptoms categorized in the Ulm SPATZ health study

	Time		*Total*		*Child sex: male*		*Child sex: female*	
			n/N	%	n	%	n	%
**Maternal depression symptoms (HADS-D)**^a^	0 M	no symptoms	576/595	96.8	288	95.7	288	98.0
		mild symptoms	16/595	2.7	10	3.3	6	2.0
		moderate/severe symptoms	3/595	0.5	3	1.0	-	-
	6 M	no symptoms	538/573	93.9	271	93.5	267	94.4
		mild symptoms	27/573	4.7	16	5.5	11	3.9
		moderate/severe symptoms	8/573	1.4	3	1.0	5	1.8
	12 M	no symptoms	535/573	93.4	270	94.1	265	92.7
		mild symptoms	27/573	4.7	13	4.5	14	4.9
		moderate/severe symptoms	11/573	1.9	4	1.4	7	2.5
**Maternal anxiety symptoms (HADS-A)**^a^	0 M	no symptoms	499/594	84.0	250	83.0	249	85.0
		mild symptoms	71/594	12.0	38	12.6	33	11.3
		moderate/severe symptoms	24/594	4.0	13	4.3	11	3.8
	6 M	no symptoms	483/573	84.3	238	82.1	245	86.6
		mild symptoms	66/573	11.5	39	13.5	27	9.5
		moderate/severe symptoms	24/573	4.2	13	4.5	11	3.9
	12 M	no symptoms	476/573	83.1	239	83.3	237	82.9
		mild symptoms	69/573	12.0	38	13.2	31	10.9
		moderate/severe symptoms	28/573	4.9	10	3.5	18	6.3

*0 M* Delivery, *6 M* 6 months, *12M *12 months

^a^no symptoms/normal: < 8, mild symptoms: ≥ 8–10, moderate/severe symptoms: ≥ 11 points

**Fig. 2:**
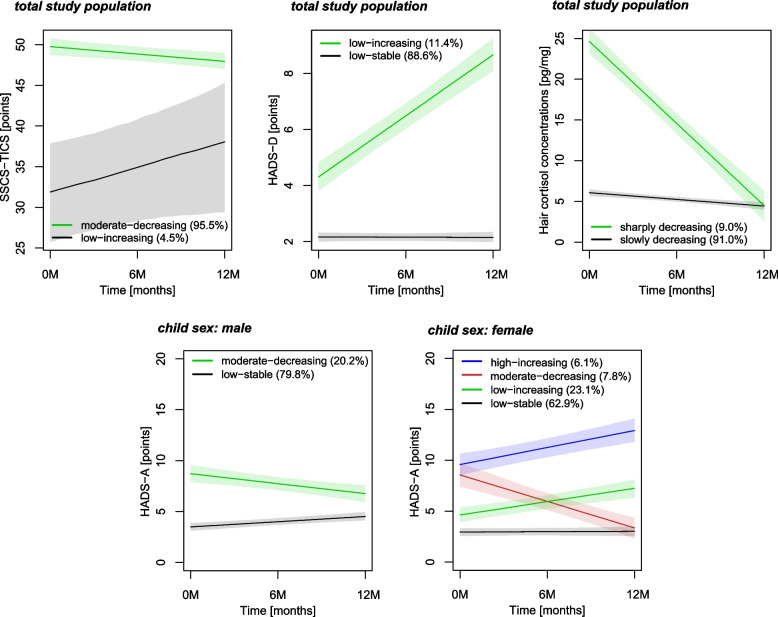
Identified trajectories of maternal stress and related symptoms and trajectories of hair cortisol concentrations. SSCS-TICS Maternal chronic stress, HADS-D Maternal depression symptoms, HADS-A Maternal anxiety symptoms, 0 M Delivery, 6 M 6 Months, 12 M 12 Months

With respect to SSCS-TICS and HADS-D trajectories from 0 to 12 M, we identified two groups each (see Fig. 2). A vast majority of mothers (95.5%) scored moderately on SSCS-TICS at 0 M and showed a decreasing trajectory over time. In contrast, 4.5% of mothers started low but with increasing SSCS-TICS scores. Furthermore, 11.4% showed low but increasing, 88.6% low-stable depression symptoms. The trajectories of SSCS-TICS and HADS-D were similar across child sexes. Conversely, we identified four anxiety trajectory groups, a low-stable group (62.9%), a low-increasing (23.1%), a moderate-decreasing (7.8%), and a moderate-increasing group (6.1%) in girls. In boys however, only two groups were observed. For trajectories of HCC, we detected a sharply decreasing (9.0%) and a slowly decreasing group (91.0%).

In adjusted models, low-increasing anxiety symptoms trajectories were positively associated with child BMI at age 3 years (b = 0.58, 95% CI: 0.11; 1.04) when compared to the low-stable trajectory group. These associations were restricted to girls and slightly lower with regard to BMI-SDS (b = 0.41; 95% CI: 0.10; 0.73).We did not find respective statistically significant associations between trajectories of maternal chronic stress, depression symptoms, or hair cortisol concentrations and child BMI.

Results compared well with the complete case model (without imputation) for BMI and BMI-SDS when maternal variables were considered as confounders. However, when introducing paternal education, the association between low-increasing anxiety and child weight in girls was attenuated to non-significance in the complete case model (BMI: b = 0.34, 95% CI: -0.29; 0.98; BMI-SDS: 0.27, 95% CI: -0.18; 0.71).

## Discussion

Given three time points of maternal stress assessment, measurement of depression and anxiety symptoms, and maternal hair cortisol concentrations from delivery to child age 3 years, we identified trajectories of (markers of) maternal psychosocial stress and of anxiety and depression symptoms in 596 mothers and their associations with child BMI at age 3 years. The trajectory of low-increasing maternal anxiety symptoms was statistically significantly associated with higher child BMI or BMI-SDS in contrast to low-stable maternal anxiety symptoms in girls. Trajectories of maternal depression symptoms were not associated with child BMI/BMI-SDS and we observed no associations of maternal chronic stress trajectories or HCC with child BMI.

### Maternal psychosocial stress

A null-effect of maternal stress on child BMI/BMI-SDS is partly in line with two recent reviews, [10, 11] as the authors argued that the two main types of stressors that were indeed associated to subsequent child overweight or obesity were environmental stressors [10, 11] and mother–child dysfunctional relationship [10]. Both types of stress are not specifically targeted by the SSCS-TICS questionnaire. In one more recently conducted longitudinal study [26] however, including *n* = 9206 mother–child dyads, maternal psychosocial stress at 5 years of age predicted child overweight and obesity at 11 years. Yet, accounting for early factors as well as sociodemographic, dietary or physical activity attenuated this association to statistical non-significance. Furthermore, postnatal maternal stress during the first year after birth had a positive longitudinal relationship with children’s BMI z-scores up to the age of five years [27] but sex-stratified analyses revealed that only girls showed this positive association while boy’s BMI z-scores were unaffected by maternal stress. It should be noted that our study might be underpowered to show statistically significant differences as it comprises a high proportion of mothers with relatively low stress levels. Interestingly, findings for the relationship between maternal stress and children's weight-related behaviours showed no evidence for an association with children's unhealthy dietary intake, but for an association of maternal stress with children's lower physical activity and higher sedentary behaviour [28]. Further studies including these variables allowing sex-specific analyses would be desirable.

### Maternal depression symptoms

According to a review, [29] a stable moderate-high or high symptom trajectory of depression was reported in most studies on perinatal depression trajectories, suggesting a high-risk group with persistent depression symptoms. Accordingly, chronic but not episodic maternal depression is associated with a greater risk for child overweight [12]. We did not find such a group but a low-stable and a low-increasing depression symptom group. However, our results concerning the association with the outcome variable are in line with further studies [13, 14] which did not find consistent associations between (multiply assessed) depression symptoms and BMI or BMI z-scores. Instead, antenatal and postpartum depressive symptoms were partly differentially associated with the outcome.

### Maternal anxiety symptoms

Few studies have examined maternal anxiety trajectories [30] and data on anxiety symptoms and child weight are scarce. We found higher child BMI with mothers indicating low-increasing compared to low-stable anxiety symptoms in girls which may suggest a higher impact of more recent as opposed to earlier experiences of anxiety symptoms on child BMI. The positive association between high-increasing anxiety trajectory and child BMI would correspond to this assumption. However, also moderate-decreasing maternal anxiety symptoms are positively associated with higher child BMI in girls (see Table 4). These results are contradictory and need further explanation. Due to the relatively small sample size, our results do certainly need confirmation in large scaled studies. Especially since Shay et al. 2020 [31] observed no direct relationship between maternal anxiety and child overweight but both variables were associated with decreased weeks of breastfeeding, where we did not find a respective association. Notably, the authors analyzed clinically significant anxiety symptoms which were not highly prevalent in our study.

**Table 4:** Associations of trajectories of maternal chronic stress, depression and anxiety symptoms, and hair cortisol concentrations with child BMI/BMI-SDS in the Ulm SPATZ health study

		**Child BMI**^a^		**Child BMI-SDS**^b^	
	**n**	**Regression coefficient**	**95% Confidence interval**	**Regression coefficient**	**95% Confidence interval**
**Maternal chronic stress (SSCS-TICS)**					
Low-increasing	569	0	-	0	-
Moderate-decreasing	27	-0.41	-1.00; 0.18	-0.24	-0.65; 0.16
**Maternal depression symptoms (HADS-D)**					
Low-stable	528	0	-	0	-
Low-increasing	68	-0.03	-0.38; 0.32	-0.03	-0.26; 0.21
**Maternal anxiety symptoms (HADS-A)**					
**Child sex: female**					
Low-stable	185	0	-	0	-
High-increasing	18	0.40	-0.25; 1.05	0.21	-0.23; 0.66
Moderate-decreasing	23	0.20	-0.42; 0.82	0.17	-0.25; 0.60
Low-increasing	68	**0.58**	**0.11; 1.04**	**0.41**	**0.10; 0.73**
**Child sex: male**					
Low-stable	241	0	-	0	-
Moderate-decreasing	61	0.04	-0.34; 0.43	-0.00	-0.26; 0.26
**Maternal hair cortisol concentrations**					
Slowly decreasing	535	0	-	0	-
Sharply decreasing	53	-0.24	-0.75; 0.07	-0.24	-0.51; 0.04

*BMI* Body mass index, *BMI-SDS* Age- and sex-standardized BMI, bold letters indicate statistically significant associations

^a^adjusted for maternal BMI at first detection of pregnancy, child birth weight, maternal education, maternal smoking in the year before pregnancy, child sex (not for HADS-A), child age at BMI measurement, paternal education, and paternal BMI

^b^adjusted for maternal BMI at first detection of pregnancy, child birth weight, maternal education, maternal smoking in the year before pregnancy, child age at BMI measurement, paternal education, and paternal BMI

### Hair cortisol concentrations

After excluding extremely high cortisol concentrations as suggested by Marceau et al. 2020 [21], we found no association between maternal HCC and child weight. It is well known that during pregnancy, cortisol concentrations are about 3-fold higher compared to a non-pregnant state. Little is known however about the post-pregnancy period and HCC “return to normal” or whether the different trajectories of peripartum HCC reflect different stress or anxiety or depression trajectories. In their review Meyer et al. 2021 [32] concluded that the current literature lacks consistent evidence that HCC are related to chronic stress, anxiety or depression symptoms. This finding is also supported by our group [33, 34] and by Spearman correlation coefficients ≤ 0.09 when analyzing the associations between chronic psychosocial stress, depression or anxiety symptoms and HCC at 6 M and 12 M.

Our study design allowed to identify trajectories of maternal psychosocial stress and related symptoms following delivery and thus may address chronicity of stress exposure or anxiety/depression symptoms. Yet, a possible selection bias due to loss to follow-up of non-German, less educated or larger families might impact the results as experiencing stress may cluster with social and economic adversities. Thus, the real magnitude of the association might be underestimated in our study. Further variables such as birth mode, seasonality, batch of hair cortisol sample (see e.g., [17, 21]) might influence trajectories of hair cortisol concentrations. Larger studies are yet needed to analyze the respective subsamples and their trajectories as well as their associations with child weight. Interestingly, the prevalence of children who suffer from overweight or obesity is slightly lower in our study compared to the general population in Germany, where 10.8% (95% CI: 7.0–16.5) of girls and 7.3% (4.7–11.1) of boys aged 3 to 6 years were either overweight or obese [35]. Unfortunately, our study provides no data to elucidate a mechanism between depression/anxiety symptoms or maternal stress and child BMI.

## Conclusions

Taken together, the results of studies analyzing the impact of maternal stress and related symptoms on child BMI are controversial. We found an association of the trajectory of maternal anxiety symptoms with child BMI/BMI-SDS at age 3 years, particularly in girls. However, further studies should address trajectories of stress or related constructs, potential mediators, and confounders such as breastfeeding behaviours as well as sex-specific differences.

## Data Availability

The datasets generated and analyzed during the current study are not publicly available due to limitations of ethical approval but are available from the corresponding author on reasonable request.

## Abbreviations

0 M: Delivery
6 M: 6 Months postpartum
12 M: 12 Months postpartum
b: Regression coefficient
BIC: Bayesian Information Criterion
BMI: Body mass index
BMI-SDS: Age- and sex-standardized body mass index
CI: Confidence interval
HADS: Hospital Anxiety and Depression Scale
HCC: Hair cortisol concentrations
IOTF: International Obesity Task Force
P: Percentile
r: Spearman correlation coefficient
SSCS-TICS: The Screening Scale of the Trier Inventory of Chronic Stress
WHO: World Health Organization
